# Supplementary material for: Identification of photoperiod-induced specific miRNAs in the adrenal glands of Sunite sheep (Ovis aries)
Source: Front Vet Sci. 2022 Jul 22;9:888207. doi: 10.3389/fvets.2022.888207 (PMC9354845; doi:10.3389/fvets.2022.888207)
Supplement: Supplementary file 1 [file Data_Sheet_1.ZIP › Supplementary file/miRDeep2 file.zip!/miRDeep2 file/Novel_Structure/SPLP42A2/Novel_676.pdf]

Provisional ID	:	NC_019461.2_13477
Score total	:	9.7
Score for star read(s)	:	3.9
Score for read counts	:	1.7
Score for mfe	:	2.5
Score for randfold	:	1.6
Score for cons. seed	:	
Total read count	:	15
Mature read count	:	9
Loop read count	:	4
Star read count	:	2

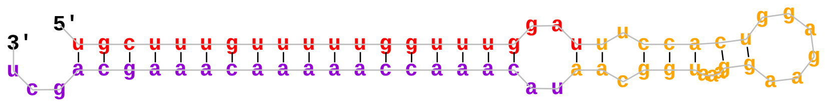

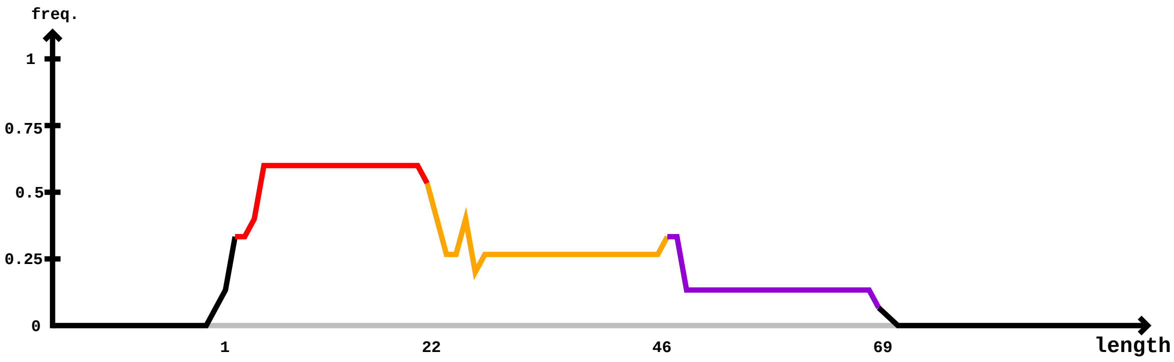

Mature Star

```
5'- cgggcgggguuauuagcugcuuuguuuuugguuuggauuuccacuggagaaggaaauggcaauacaaaccaaaaacaaagcagcuaaaauaaccccgccuguuuagaaugau   -3'    obs
    cgggcgggguuauuagcugcuuuguuuuugguuuggauuuccacuggagaaggaaauggcaauacaaaccaaaaacaaagcagcuaaaauaaccccgccuguuuagaaugau          exp
    (((((((((((((((((((((((((((((((((((..((.(((((......))...))).))..))))))))))))))))))))))))..)))))))))))...........   reads   mm   sample
    ...............gcugcuuuguuuuugguuugga...........................................................................   1       0    seq
    ................cugcuuuguuuuugguuuggauu.........................................................................   1       0    seq
    .................ugcuuuguuuuugguuuggau..........................................................................   2       0    seq
    .................ugcuuuguuuuugguuuggauu.........................................................................   1       0    seq
    ...................cuuuguuuuugguuuggauuucc......................................................................   1       0    seq
    ....................uuuguuuuugguuuggauuucc......................................................................   2       0    seq
    ....................uuuguuuuugguuuggauuucca.....................................................................   1       0    seq
    .........................................cacuggagaaggaaauggcaa..................................................   1       0    seq
    .........................................cCcuggagaaggaaauggcaaua................................................   1       1    seq
    ...........................................cuggagaaggaaauggcaaCa................................................   2       1    seq
    ..............................................................uacaaaccaaaaacaaagcagc............................   1       0    seq
    ..............................................................uacaaaccaaaaacaaagcagcu...........................   1       0    seq
```
